# Editorial: The cognitive neuroscience of visual working memory, Volume II

**DOI:** 10.3389/fnsys.2022.1017754

**Published:** 2022-09-14

**Authors:** Natasha Sigala, Zsuzsa Kaldy, Greg D. Reynolds

**Affiliations:** ^1^Brighton and Sussex Medical School, University of Sussex, Brighton, United Kingdom; ^2^Department of Psychology, University of Massachusetts, Boston, MA, United States; ^3^Department of Psychology, The University of Tennessee, Knoxville, TN, United States

**Keywords:** working memory, cognition, vision, neuroscience, computational modeling

Visual Working Memory (VWM) enables us to retain and manipulate visual information for a short amount of time in order to perform a specific task. Following on from a popular volume on the topic published in 2017 (Sigala and Kaldy, 2017), we embarked on editing an updated collection 5 years later, to capture a snapshot of the current research in the field. With almost 4,000 studies published in the last 5 years on visual working memory, it is extremely difficult to provide a comprehensive overview of the topic. However, the articles that form this collection cover considerable ground in the field.

In a computational study, Matsumoto et al. investigate the effect of simulated recurrent connections in a model that combined the properties of a deep neural network (DNN), and a Recurrent Neural Network RNN). DNNs have been very successful on computer vision tasks, but at the same time they are complex and non-linear, presenting considerable issues of interpretation and reliability (e.g., Samek et al., 2021). Matsumoto compared the Xception net with a Hopfield model, an associative memory model that the authors have previously shown to behave similarly to TE neurons in the Inferior Temporal (IT) cortex (Matsumoto et al., 2005). They found that the combined model performed better than the DNN alone in a hierarchical categorization task, and they suggest that the fully connected layers in the Hopfield model represent the Prefrontal Cortex (PFC). The IT and prefrontal cortices are both active in tasks that involve object recognition and categorization (e.g., Kar and DiCarlo, 2021), so this suggested neural architecture is a plausible model for the VWM element of hierarchical categorization.

In another study employing RNNs, Xie et al. put to the test the assumption of the Standard Model of WM that PFC neurons behave like a continuous attractor that maintains information in spiking activity, also referred to as “bump attractor” models (e.g., Jaffe and Constantinidis, 2021). They trained the RNNs on an Oculomotor Delayed Response (ODR) task, and one with a Distractor (ODRD). The RNN connectivity footprint emerges through training, while in bump attractor models it is hardwired according to the tuning of connected units. Despite this difference, the RNN output resembled that of the bump attractor models, and was closer to the activity of actual neurons, including RNN units with different stimulus preferences during different task periods (e.g., Sigala et al., 2008). Importantly, as the authors state, the study is also compatible with activity-silent (non-spiking) accounts of WM, which could involve changes in synaptic weights or dynamic encoding (e.g., Stokes et al., 2013; Erez et al., 2022), or indeed rhythmic discharges, as explored in the next paper in this collection (Rezayat et al.).

Rezayat et al. present a review of the literature on coordinated brain activity during WM, including behavioral performance correlates, causal interventions and breakdown of oscillatory activity in mental illness. The review incorporates evidence from non-human primates, including recordings of single units and Local Field Potentials (LFPs), as well as studies of human intracranial recordings (ECoG). The authors present evidence which supports the idea that the delay activity of individual neurons often fails to predict WM performance, while population-level signatures (such as LFPs) are more likely to predict it successfully. They then move on to examine the conceptual models of synchronized activity between the PFC and other brain areas during WM, including sensory cortex (sensory recruitment), parietal cortex (distributed networks), and the hippocampus (activation of long-term memory). After a summary of brain disorders where oscillations and synchrony in WM break down (including psychosis, autism, and depression, among others), they survey the effects of manipulating inter-areal synchrony on WM performance for a variety of stimulation methods. Finally, they propose a novel framework with a specific role for synchrony in the function of VWM (Figure 3 in Rezayat et al.). This involves a top-down modulation of spike timing in visual areas from the PFC, along with a shared oscillatory frame of reference that allows sensory stimuli that match the WM contents to be more likely to drive PFC activity and behavior.

Finally, Assecondi et al. have contributed an experimental study that combined a 3-day WM training with tDCS (transcranial Direct Current Stimulation), and looked for changes in event-related potentials (ERPs) 1 day and 1 month after the training in healthy young adults. The particular tDCS protocol did not result in behavioral or brain activity changes in this participant sample, however the area of cognitive training, and promoting brain plasticity with tDCS remain popular, and limitations as well as future directions are discussed.

In summary, this Research Topic includes two computational papers, an empirical study of WM training, and a narrative review. They all present evidence in favor of an integrative and multipronged approach that will help elucidate the mechanisms of WM in health and disease, and offer a snapshot of the exciting current work and theories in the field.

## Author contributions

All authors listed have made a substantial, direct, and intellectual contribution to the work and approved it for publication.

## Conflict of interest

The authors declare that the research was conducted in the absence of any commercial or financial relationships that could be construed as a potential conflict of interest.

## Publisher's note

All claims expressed in this article are solely those of the authors and do not necessarily represent those of their affiliated organizations, or those of the publisher, the editors and the reviewers. Any product that may be evaluated in this article, or claim that may be made by its manufacturer, is not guaranteed or endorsed by the publisher.
